# Specification of Neck Muscle Dysfunction through Digital Image Analysis Using Machine Learning

**DOI:** 10.3390/diagnostics13010007

**Published:** 2022-12-21

**Authors:** Filip Paskali, Jonathan Simantzik, Angela Dieterich, Matthias Kohl

**Affiliations:** 1 Institute of Precision Medicine, Medical and Life Sciences, Hochschule Furtwangen, 78054 Villingen-Schwenningen, Germany; 2 Physiotherapie, Fakultät Gesundheit, Sicherheit, Gesellschaft, Hochschule Furtwangen, Studienzentrum Freiburg, 79110 Freiburg, Germany

**Keywords:** neck pain, shear wave elastography, ultrasound, image analysis, machine learning

## Abstract

Everyone has or will have experienced some degree of neck pain. Typically, neck pain is associated with the sensation of tense, tight, or stiff neck muscles. However, it is unclear whether the neck muscles are objectively stiffer with neck pain. This study used 1099 ultrasound elastography images (elastograms) obtained from 38 adult women, 20 with chronic neck pain and 18 asymptomatic. For training machine learning algorithms, 28 numerical characteristics were extracted from both the original and transformed shear wave velocity color-coded images as well as from respective image segments. Overall, a total number of 323 distinct features were generated from the data. A supervised binary classification was performed, using six machine-learning algorithms. The random forest algorithm produced the most accurate model to distinguish the elastograms of women with chronic neck pain from asymptomatic women with an AUC of 0.898. When evaluating features that can be used as biomarkers for muscle dysfunction in neck pain, the region of the deepest neck muscles (M. multifidus) provided the most features to support the correct classification of elastograms. By constructing summary images and associated Hotelling’s T^2^ maps, we enabled the visualization of group differences and their statistical confirmation.

## 1. Introduction

Almost everyone has or will have experienced some degree of neck pain [1]. Most neck pain is unspecific with the tendency to become recurrent [2]. The one-year prevalence of disabling neck pain has been estimated between 1.7% and 11.5% [3], and the risk is especially high in middle-aged women who have already experienced neck pain [2]. Typically, neck pain is associated with the sensation of tense, tight, or stiff neck muscles [4]. Therapeutic interventions often include measures to reduce neck muscle tone. However, it is unclear whether the neck muscles are objectively stiffer with neck pain. Some investigations documented higher stiffness of the neck muscles with neck pain [5,6], while others did not find differences compared to asymptomatic study participants [7,8]. Knowledge of a potentially increased objective stiffness of the neck muscles is important when related to diagnosis or therapeutic decisions [9].

Currently, shear-wave elastography is the superior modality for measurements of tissue stiffness. Ultrasound shear wave elastography (SWE) is more precise than muscle hardness meters [10] and enables reliable measurements of neck muscle stiffness [11,12]. However, the methods to measure muscle stiffness vary between studies. Muscle stiffness is usually not homogeneous but varies locally. Most SWE systems provide a region of interest (ROI) for the measurements that is much smaller than the visible area of the muscle. The active and individual positioning of the ROI has a biasing potential. Some systems allow for the manual circumscription of a visually determined muscle region to include a large muscle area. Often, the elastograms include some black areas in which the shear waves could not be tracked. These areas affect the measurements and must be avoided, which again reduces the included measured muscle area. It can be assumed that the inclusion of all pixels with sufficient quality over the full visible muscle area provides the most representative stiffness measurements for the respective muscle.

Although some research has been carried out on investigating neck muscle stiffness, only one study [7] has attempted to investigate muscle stiffness differences in the deep neck muscles between chronic neck pain and asymptomatic patients. Inclusion of the deep neck muscles may be of relevance because the superficially accessible trapezius muscle primarily controls scapular movement [12]. Moreover, evidence supports differences in the activation of the deep neck muscles with neck pain [13,14]. Several studies support altered and less variable activation of the deep-lying spinal muscles in chronic spinal pain [15,16,17].

New technologies have become a standard tool to address diverse research questions in broad contexts in medical diagnosis. This covers various methods, e.g., utilizing virtual reality to detect motion sickness in Parkinson’s disease patients [18], or using machine learning to reason about aging and a healthy lifestyle based on soft tissue measurements [19]. Specifically, the usage of artificial intelligence algorithms in image analysis has seen a growing occurrence in recent publications.

Machine learning algorithms have been employed, for example, for the classification of chronic liver diseases [20], breast tumors [21], thyroid nodules [22], prostate cancer [23] and knee cartilage degeneration [24]. On the other hand, deep learning convolutional neural network algorithms have been used for the segmentation of muscles as well as the automatic measurement of muscle thickness and muscle fat infiltration [25,26,27]. To the best of our knowledge, there have been no attempts to employ machine learning algorithms in ultrasound elastography images for the classification of neck pain. Moreover, such an automated approach enables a computed image analysis, which may provide new insights into differences in the mechanical properties of the neck muscles in individuals with neck pain compared to asymptomatic individuals. The study presented here extends the analysis carried out by Dieterich et al. in 2020 and proposes the application of modern image analysis and machine learning methods considering different aspects of the stiffness areas in the neck muscles to investigate the changes of objective muscle stiffness with chronic neck pain detectable in 2-D ultrasound elastography.

The primary objective of this research was to train a machine learning model that by analyzing elastography shear wave images from the neck area can objectively predict neck muscle dysfunction with high accuracy. The secondary aim was to examine the spatial disposition of the stiffness difference in the neck extensor muscles in the active and resting state between women with and without chronic neck pain.

The remainder of the paper is organized into four additional distinct sections. The second section is concerned with the materials and methodology employed for this study. In the third section, we present the results. In the fourth section, we explain our findings in more detail and compare them with the findings of other studies. The final section consists of a summary of the study’s findings.

## 2. Materials and Methods

The current study used ultrasound elastography images (elastograms) from a cross-sectional, observational study undertaken by Dieterich et al. in 2020 [7]. The original study has been approved by Medical Ethics Committee of the Medical Center Göttingen, Germany (No.23/10/15). Participants provided written informed consent prior to study participation, and all procedures were performed in accordance with the Declaration of Helsinki. The elastograms were obtained from 38 adult women, 20 with chronic neck pain and 18 asymptomatic. The inclusion criteria for the pain group were non-specific pain longer than six months with symptom duration of at least one week, neck stiffness sensation, and a Neck Disability Index higher than 10/50. On the other hand, for the control group, inclusion criteria included no history of recurrent neck or low back pain or neck pain that affected the neck function or required treatment. Exclusion criteria were major circulatory, neurological, or respiratory disorders, pregnancy, cervical spinal surgery, participation in neck muscle training in the past 6 months, body mass index higher than 30, and intake of medications that may affect muscle stiffness. On the days of imaging, participants were asked to not use pain medication. As shown in the demographic summary (Table 1), the sample size, age, and body mass index of the two groups are comparable. The range of motion in flexion, extension, and maximal voluntary isometric contraction is reduced in the pain group.

During the imaging sessions, participants performed 14 diverse activities including a head lift from prone, stressful office work, they balanced a weight (1 kg) on the head and graded isometric neck extension at force levels of 12 N, 24 N, 36 N, and 48 N. The activities were repeated thrice, in three different imaging sessions. Overall, this would lead to (20 + 18) × 14 × 3 = 1596 images. However, not all participants were able to perform a complete set of activities during each imaging session, and images from four (two control and two pain) participants had to be removed due to incomplete clinical data. During the quality control, eight images with a missing quality map were excluded from the analysis. The images have been recorded from 34 (16 control–18 pain) participants in a median of 14 (IQR: 13–14) images per participant in the first session, 11 (IQR: 10–11) images in the second session, and nine (IQR: 8–9) images in the third session. In total, 1099 images (512 control-587 pain), 454 from the first session, 356 images from the second session, and 289 images from the third session were useable for the image analysis.

Elastograms were obtained on the neck extensor muscle group 1 cm lateral to the spinous processes, the transducer centered at the C4 level in longitudinal orientation (Acuson S3000; Siemens, Germany, 9L4 linear transducer with 4 cm footprint). A maximal shear wave speed of 10m/s was set. Gain (14–20 dB), dynamic range (45–65 dB), and image depth 3.5–4.5 cm were adjusted for good visualization.

Each measurement produced three images, a B-mode ultrasound image; a color-coded elastogram indicating the shear wave velocity in the ROI with a range of 0.5–10 m/s; and a color-coded quality map presenting the quality of shear wave detection in each pixel ranging from low quality to high quality. The images have a resolution of 1024 × 768 and were stored in BMP file format. For the data analysis, we categorized the images according to functional differences of the performed tasks. We distinguished a low force and a high force category. The low force category consisted of images obtained while the participants were in a resting state or performing neck extension with a maximal force level of 12 N, whereas the high force category consisted of images obtained while the participants performed neck extension at levels of 36 N and 48 N. Alternatively, images were categorized according to tasks that require precise muscle activation (targeting a specific force level, balancing task) and less precise muscle activation (office work and prone neck extension).

The image analysis was carried out using the free open-source programming language Python (3.10) [28]. The packages used in the analysis are: Numpy (1.23.4) [29] and Scipy (1.9.3) [30] to extend the Python core functionally for scientific computing; Matplotlib (3.6.2) [31] for figure generation and visualization; Pandas (1.3.5) [32] for producing and the manipulation of data tables; Pillow (9.3.0) [33] and Scikit-Image (0.19.3) [34] for image processing; and Scikit-Learn (1.1.3) [35] for machine learning.

**Figure 1:**
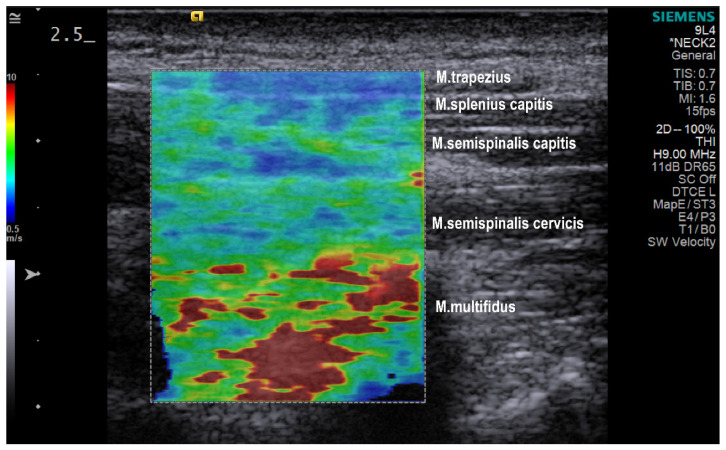
Color-coded elastography shear wave image with muscle layer organization.

For the training of the machine learning algorithms, 28 features were handcrafted, extracting numeric information from the shear wave velocity color-coded images (Table 2). The first set of numerical features was computed by performing various statistical operations on the whole image, such as computing grand mean, median, and standard deviation. Furthermore, the images were separated into three color channels: red, green, and blue. The statistical operations and grand sum were computed for each color channel of each image. The images were converted into grayscale color mode and the same features were extracted. For the second set of features, an inverse mapping was performed from RGB color mode to stiffness using the full range shear velocity scale (Figure 1). The number of pixels in the images was quantified from three stiffness level categories: low-level stiffness category with a shear velocity between 0.5–3.67 m/s, medium-level stiffness category with a shear velocity between 3.67–6.84 m/s, and high-level stiffness category with a shear velocity between 6.84–10 m/s. The third set of features was obtained with Gabor filtering, which was used in several studies for texture classification [36,37,38]. In this study, we used the mean and variance of the resulting convoluted image as features, using Gabor kernels with four different angles: 45°, 90°, 135° and 180°. Finally, the images were divided into ten equal horizontal segments and the aforementioned operations were repeated for each of the segments leading to a total number of 308 features. To the 308 features extracted from each shear wave elastography image, 14 activities performed by the participants were added as 13 dummy variables in order to investigate the impact of different physical activities on the differences between both groups. Additionally, to investigate the technical variability, as a negative control, two dummy variables were added, representing the three imaging sessions. Overall, this gives a total of 323 features extracted from each of the 1099 images.

**Figure 2:**
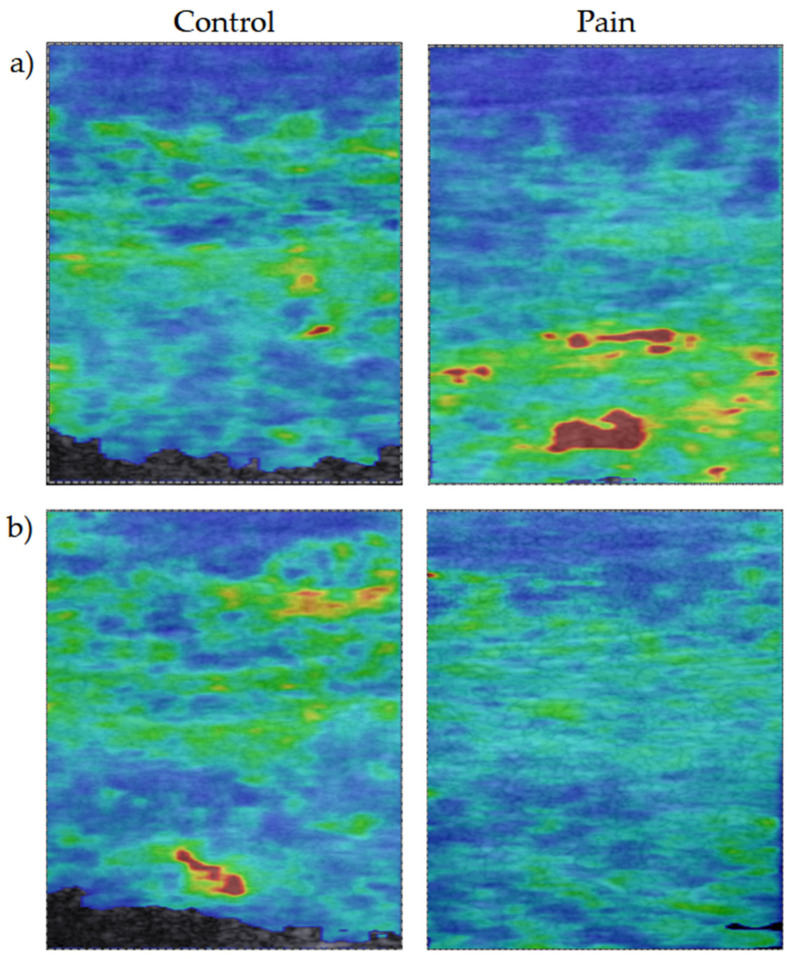
A sample of shear wave elastography images from the control and pain group, recorded during neck extension while lying prone; (**a**) The difference between classes is visually detectable, with increased stiffness in the bottom part of the image from the pain group; (**b**) The assigning to both classes by visual inspection is challenging.

**Figure 3:**
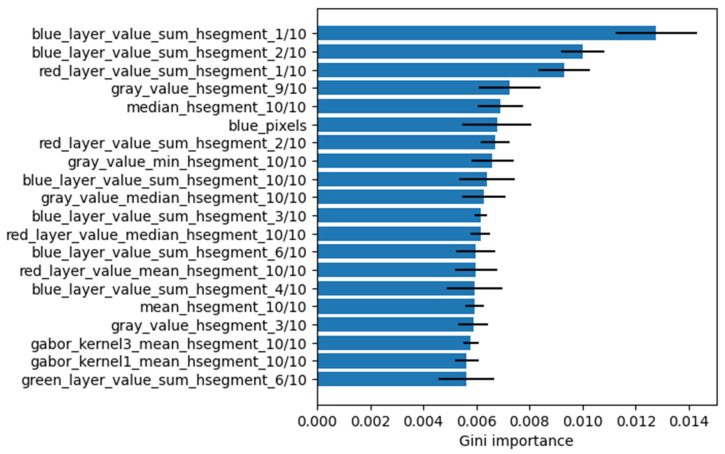
Top 20 most important features extracted from trained Random Forest model, sorted by Gini importance. The names of the features are explained in more detail in the supplement Table S1.

A supervised binary classification was performed, using six classification machine learning algorithms from the package scikit-learn [35], as follows: Random Forest (RF) [39], Decision Trees (DT) [40], Support Vector Machines (SVM) [41], K Nearest Neighbor (KNN) [42], Logistic Regression (LR) [43] and Naive Bayes (NB) [44]. The main task was a prediction of one of the two target classes: participants with neck pain and participants without neck pain. The class label was assigned to the images before the machine learning. Figure 2 depicts two pairs of elastograms used for the training of machine learning models. For model training and validation, we carried out nested 10-fold cross-validation with the whole dataset [45]. The model training and validation were repeated 10 times with new hyperparameter optimization using a grid search algorithm and using new ten random folds. Finally, with an intention to find reliable biomarkers that can be used to objectify and enhance future diagnostics, we extracted the features used for training the RF model and sorted them by importance (Figure 3).

**Figure 4:**
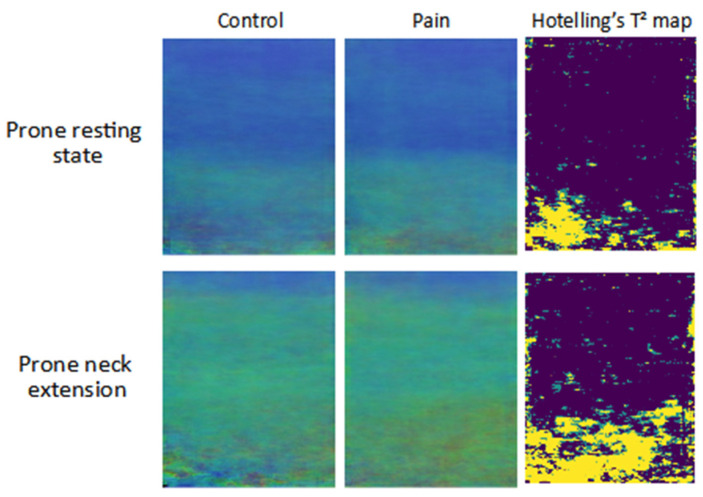
Summary mean images of the elastograms’ RGB values comparison between resting and active state of prone neck extension; Hotelling’s T^2^ test map—green color *p*-value < 0.05—yellow color *p*-value < 0.01.

**Figure 5:**
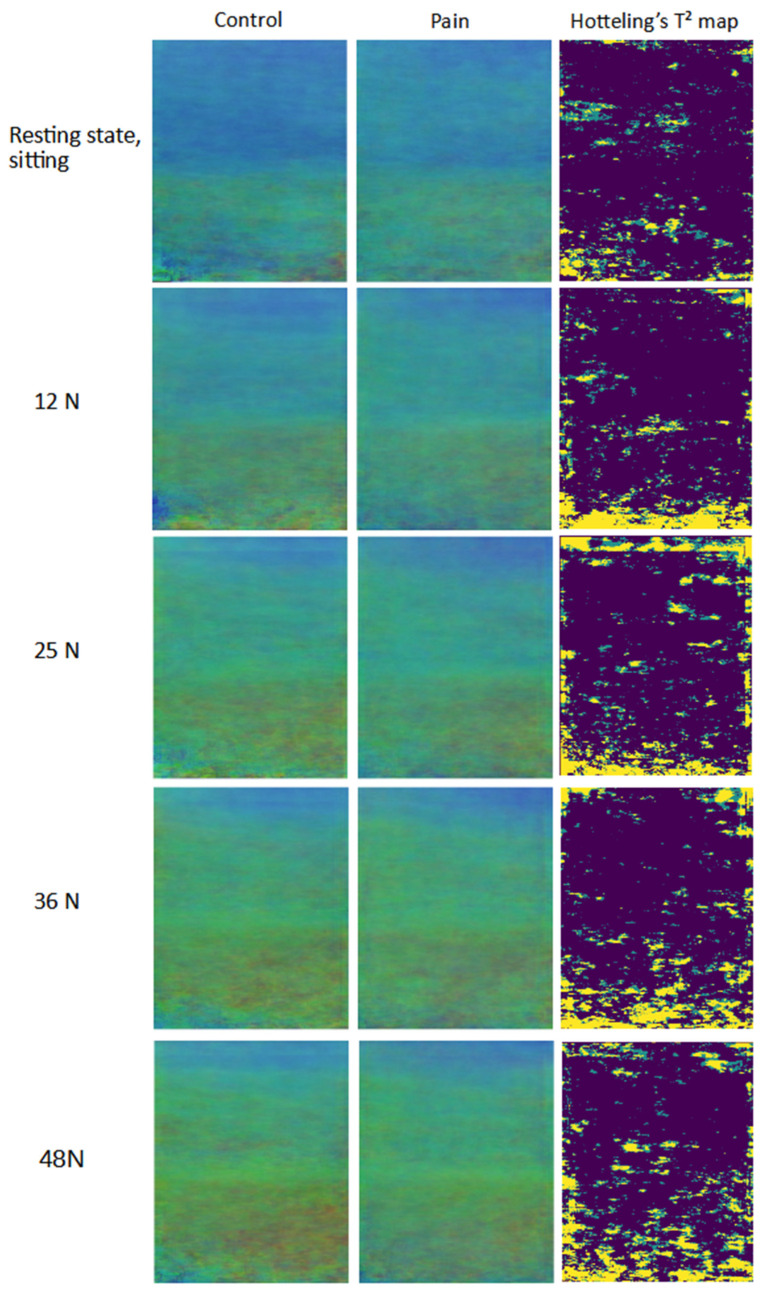
Summary mean images of the elastograms’ RGB values of the neck muscles with graded neck extension; Hotelling’s T^2^ test map—green color *p*-value < 0.05—yellow color *p*-value < 0.01. In participants with neck pain, we note higher stiffness in the upper half of the image (superficial muscles) during the resting state in sitting, and less stiffness in the lower half of the images (deep muscles) during active neck extension.

To comprehensively explore the spatial disposition of the stiffness differences between the two groups, summary images were produced, and visual analysis was carried out (Figure 4 and Figure 5). For quality control, the quality maps generated during the imaging sessions were used and pixels with a quality lower than 50% on the quality map were excluded. The summary images were generated by calculating the arithmetic mean pixelwise on the intersection of the region of interest of all images in each group. Summary images retain the color coding of the original SWE images. Additionally, they were generated for the different categories of activities performed by the participants. Therefore, they provide a visual overview for each group and enable a visual comparison by depicting the average level of stiffness in each pixel per group. To statistically verify the difference between both groups, for RGB values of each pixel Hotelling’s T^2^ test, a multivariate test comparing 3-dimensional mean vectors was performed [46]. Using this method, Hotelling’s T^2^ maps were generated for each of the different categories, revealing the regions with significant differences. The map is a color-coded representation of *p*-values for each pixel, indicating three categories: *p*-value greater than 0.05, lower than 0.05, and lower than 0.01.

## 3. Results

The arithmetic mean and the confidence interval of the performance metrics, as empirical quantiles (2.5%; 97.5%), were computed from 100 cross-validation results. The RF model showed the highest mean AUC score, closely followed by KNN and SVM with the Radial Basis Function (RBF) kernel (Table 3).

A major part of the most important features extracted from the RF model are coming from the deepest horizontal segment, which corresponds to the multifidus muscle closest to the spine (Figure 3).

The summary images of both groups use the same color coding as the elastograms and enable a visual group comparison. Hotelling’s T^2^ test maps mark the regions of significant differences between the two groups.

The visual inspection of the summary images (Figure 4 and Figure 5) shows apparent group differences in the lower half of the images corresponding to the deeper neck muscles m. semispinalis cervicis and m. multifidus. The large regions of significant difference are visible on Hotelling’s T^2^ maps (Figure 4 and Figure 5) in the lower layer of the image, while the upper half of the maps represents m. semispinalis capitis, m. splenius capitis, and m. trapezius, and displays very small regions of significant difference. Summary images of the other tasks are available in the Supplementary Data.

## 4. Discussion

In the current study, we trained and evaluated machine learning models to support the diagnosis of muscle dysfunction in neck pain. The RF model as well as KNN and SVM showed a very good performance for distinguishing elastograms of women with chronic neck pain from asymptomatic women. As we are looking for biomarkers for muscle dysfunction in neck pain, we evaluated the 20 most important features from RF. Interestingly, the region of deepest neck muscle (M. multifidus) provided nine of these 20 features. By constructing summary images and associated Hotelling’s T^2^ maps, we enabled the visualization of group differences and its statistical confirmation. Each of these findings is discussed in detail in the following paragraphs.

Among the six machine learning classifiers that were assessed, the RF demonstrated the highest mean performance, reaching an AUC score of 0.898 (with sensitivity, specificity, accuracy of 78.2%, 84.0% and 81.2%, respectively). The second-best performing algorithms are KNN (accuracy 78.9%, sensitivity 79.6%, specificity 78.2% and 0.886 AUC score) and SVM with RBF kernel (accuracy 81.4%, sensitivity 80.2%, specificity 82.5%, and 0.884 AUC score). The other algorithms showed clearly lower performances. A possible explanation for these results may be the heterogeneity of the medical data, the variance introduced by the pseudo-random selection process, or overfitting due to the high number of features in the training step. In reviewing the literature, we could not find other studies that employed machine learning algorithms on SWE data to analyze neck muscle pain. The most similar approach was used in a study by Huang et al. in 2020 [22], who did a comparative analysis of five prediction models for cervical lymph node metastasis prediction. Consistent with our findings, an RF model demonstrated the highest predictive accuracy (0.889 AUC score). Another study that used an SVM model to classify SWE images of chronic liver disease reported a maximum predictive accuracy of 87.3% [20].

The most indicative features chosen from the RF algorithm are represented in Figure 3. What stands out is that many of the features are extracted from the lowest horizontal segment, which corresponds to the multifidus muscle. This suggests that a considerable amount of information for the distinction of both groups is located in the tissue of the deep neck muscles close to the spine. Contrary to our expectations, the type of activity that participants performed did not play an important role in the classification.

The secondary aim of this study was to examine the spatial disposition of the stiffness differences in the neck extensor muscles in the active and resting state. For this purpose, we generated summary images and Hotelling’s T^2^ maps for each activity performed during the imaging sessions. The summary images and Hotelling’s T^2^ maps (Figure 4 and Figure 5) indicate the presence of differences in stiffness in the neck muscle tissue between groups, which are more pronounced with active muscle recruitment. Figure 5 demonstrates the positive correlation between force intensity, stiffness levels, and difference between groups. Generally, higher muscle stiffness is correlated with higher muscle activation [47,48,49]. Other neck pain studies reported reduced activation of the deep neck muscles in the pain group [13,14], which was also observed in our summary images of the neck muscles with graded extension (Figure 5). On the contrary, this was not observed in the summary images of neck extension in prone (Figure 4). Reduced deep muscle activity might be a result of an adaptive mechanism to avoid or reduce pain sensation. It can be assumed that pain is higher with higher muscle activity, so the adaptive mechanism might be involuntary pain management. The observation of the resting and active states in prone summary images indicates higher muscle stiffness during the resting state in the pain group (Figure 4). This might be a result of a reduced ability to relax the deep neck muscles, which again might indicate a protective strategy [50]. Up to now, there has been little research that directly assesses passive stiffness in neck muscles [9]. The higher passive stiffness in the pain group suggests that chronic pain in the neck muscles influences the passive level of stiffness. The summary images of graded neck extension display the effect of the force intensity on the spatial disposition and stiffness intensity (Figure 5). The stiffness builds up, originating from the deep neck muscles, corresponding to the lower portion of semispinalis cervicis and m. multifidus. This finding was also reported by Yavuz et al. in 2015 [49]. With the increase of force, the stiffness of the superficial muscles increases, and this increase in stiffness is pronounced in the control group. A possible interpretation might be that asymptomatic participants could use the neck muscles more actively and reach higher muscle activation levels with higher force, while the participants with neck pain involuntarily limited themselves to control and minimize the painful sensations. On the other hand, the compensation or adaptation to force may lead to different patterns of activation between groups.

With regard to the research method, some limitations should be acknowledged. The data presented here was obtained from a rather small number of individuals. An absence of a feature selection step in some of the models might have led to over-fitting or may have introduced bias in the training step, resulting in a limited performance. An additional weakness is that the machine learning models were not validated with external data, which limits the generalizability of the study results.

Further work is required to confirm and validate our findings using independent data from more comprehensive cohorts including diverse genders, consisting of measurements recorded by different persons using different machines. Moreover, additional research is needed to better understand the discrepancies between both groups and to ascertain the possibility of adopting machine learning algorithms as a robust and reliable diagnostic tool for neck pain diagnosis.

## 5. Conclusions

This study highlights the potential usefulness of machine learning methods, especially the RF, KNN and SVM models, for diagnosing neck pain by analyzing SWE images. Moreover, the feature importance extracted from the RF model indicate that the deeper neck muscles contain important information for the classification. The summary images and Hotelling’s T^2^ maps illustrate the difference of muscle stiffness in passive state, which becomes more pronounced during active muscle recruitment. Neck pain is a complex condition and until now the diagnosis has been subjective, dependent on the participant’s personal sensation of pain and the diagnostic experience of the diagnostician. The feasibility to train a classification machine learning algorithm with very good accuracy provides initial tentative evidence of objective alterations in the neck muscles of patients with chronic neck pain that can be captured with shear wave elastography. Such a machine learning algorithm that can classify with an accuracy of or better than a trained diagnostician will improve medical diagnosis and therapy.

## Figures and Tables

**Table 1:** Demographic summary: mean ± SD, median (IQR). Abbreviations: MVIC, maximal voluntary isometric contraction; NRS, numerical rating scale; SD, standard deviation; IQR, interquartile range.

	Neck Pain, n = 20	Control, n = 18
Age (years)	52.5 (12.0)	48.5 (9.0)
Body Mass Index (kg/m^2^)	23.8 ± 3.2	22.2 ± 2.4
Range of Motion Neck Flexion	50.6° ± 12.4°	69.4° ± 10.8°
Range of Motion Neck Rotation (left + right)	115.5° ± 14.3°	145.5° ± 26.6°
MVIC neck extension (N)	56.3 ± 18.5	64.0 ± 19.2
Pain Today (NRS)	3.6 ± 2.2	0
Pain Duration (years)	8.0 (16.3)	-
Neck Disability Index % (scored 0%–100%)	32.5% ± 12.3%	-

**Table 2:** The description of the features used in the training of machine learning models.

Feature Name	Description
mean	Grand mean of all pixels from all color channels
median	Grand median of all pixels from all color channels
sd	Grand standard deviation of all pixels from all color channels
red_layer_value_mean	Grand mean of the intensities in the red image layer
red_layer_value_median	Grand median of the intensities in the red image layer
red_layer_value_sum	Grand sum of the intensities in the red image layer
blue_layer_value_mean	Grand mean of the intensities in the blue image layer
blue_layer_value_median	Grand median of the intensities in the blue image layer
blue_layer_value_sum	Grand sum of the intensities in the blue image layer
green_layer_value_mean	Grand mean of the intensities in the green image layer
green_layer_value_median	Grand median of the intensities in the green image layer
green_layer_value_sum	Grand sum of the intensities in the green image layer
gray_value	Grand sum of the grayscale intensities
gray_value_mean	Grand mean of the grayscale intensities
gray_value_median	Grand median of the grayscale intensities
gray_value_min	Minimum intensity in the grayscale image
gray_value_max	Maximum intensity in the grayscale image
blue_pixels	Number of low-level stiffness in the elastography image
green_pixels	Number of medium-level stiffness in the elastography image
red_pixels	Number of high-level stiffness in the elastography image
gabor_kernel1_mean	Grand mean of the convoluted image with 45° Gabor kernel
gabor_kernel1_var	Grand variance of the convoluted image with 45° Gabor kernel
gabor_kernel2_mean	Grand mean of the convoluted image with 90° Gabor kernel
gabor_kernel2_var	Grand variance of the convoluted image with 90° Gabor kernel
gabor_kernel3_mean	Grand mean of the convoluted image with 135° Gabor kernel
gabor_kernel3_var	Grand variance of the convoluted image with 135° Gabor kernel
gabor_kernel4_mean	Grand mean of the convoluted image with 180° Gabor kernel
gabor_kernel4_var	Grand variance of the convoluted image with 180° Gabor kernel
[feature_name]_hsegment_[n]/10	Naming of the features extracted from the horizontal segments; n—the horizontal segment number (1—close to the surface, 10—close to the spine)

**Table 3:** Summary of the cross-validation during training of the classification machine learning algorithms (mean, confidence interval 95%) Abbreviations: KNN—K-Nearest Neighbor; SVM—Support Vector Machine; AUC—Area Under Curve.

Metrics	KNN	Logistic Regression	Naïve Bayes	SVM	Decision Trees	Random Forest
Accuracy	0. 789 (0.721–0.863)	0.696 (0.609–0.773)	0.621 (0.540–0.690)	**0.814** (0.755–0.877)	0.708 (0.618–0.795)	0.812 (0.755–0.873)
Balanced Accuracy *	0.790 (0.719–0.863)	0.695 (0.607–0.767)	0.620 (0.537–0.689)	**0.814** (0.754–0.876)	0.707 (0.619–0.798)	0.811 (0.750–0.872)
Sensitivity	0.798 (0.701–0.890)	0.671 (0.526–0.818)	0.600 (0.455–0.736)	**0.802** (0.708–0.889)	0.689 (0.543–0.833)	0.782 (0.686–0.872)
Specificity	0.782 (0.687–0.868)	0.720 (0.612–0.824)	0.640 (0.527–0.741)	0.825 (0.744–0.907)	0.726 (0.568–0.816)	**0.840** (0.770–0.924)
AUC Score	0.886 (0.834–0.929)	0.771 (0.682–0.849)	0.682 (0.588–0.755)	0.884 (0.836–0.937)	0.707 (0.619–0.798)	**0.898** (0.842–0.946)
Brier Score	0.144 (0.112–0.174)	0.206 (0.161–0.255)	0.364 (0.297–0.451)	**0.137** (0.107–0.169)	0.292 (0.382–0.205)	0.149 (0.128–0.166)

* arithmetic mean of sensitivity and specificity; The best results are in bold.

## Data Availability

The complete Python code developed in this project is open-source and is available on GitHub (https://github.com/fpaskali/neck-swe-classification (accessed on 9 December 2022)) under L-GPL-3 license. The data that support the findings of this study are available from AD, upon reasonable request.

## Supplementary Materials

The following are available online at https://www.mdpi.com/article/10.3390/diagnostics13010007/s1.
