# Supplementary material for: Specification of Neck Muscle Dysfunction through Digital Image Analysis Using Machine Learning
Source: Diagnostics (Basel). 2022 Dec 21;13(1):7. doi: 10.3390/diagnostics13010007 (PMC9818408; doi:10.3390/diagnostics13010007)
Supplement: Supplementary file 1 [file diagnostics-13-00007-s001.zip › diagnostics-2070594-supplementary.pdf]

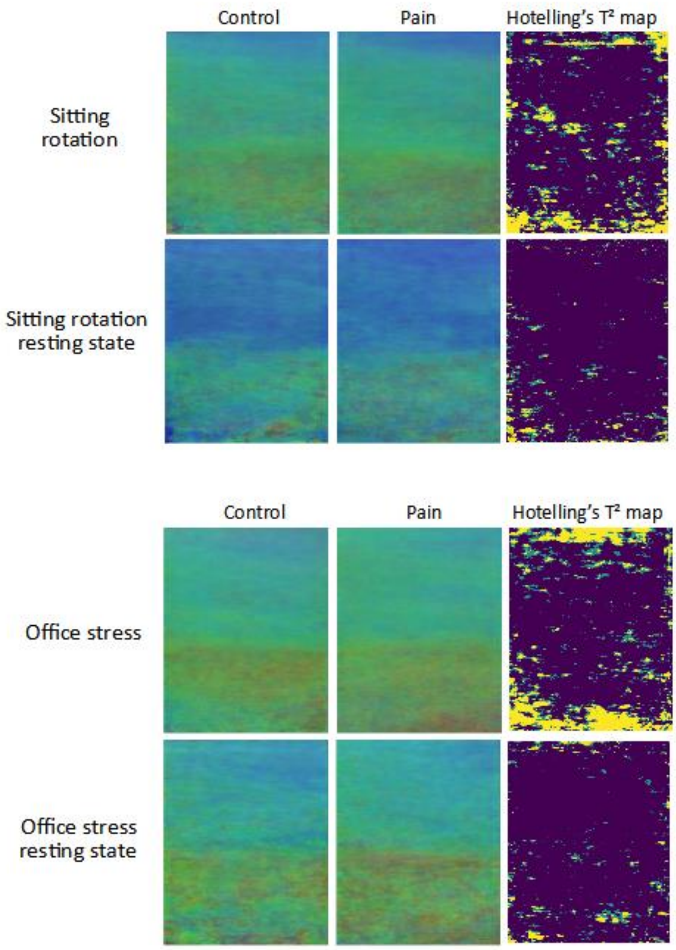

Figure S1. Summary mean images of the elastograms' RGB values; a) Comparison between neck muscle rotation and resting state while sitting; b) Comparison between office stress and resting state; Hotelling's T^2 test map – green color p-value < 0.05 – yellow color p-value < 0.01.

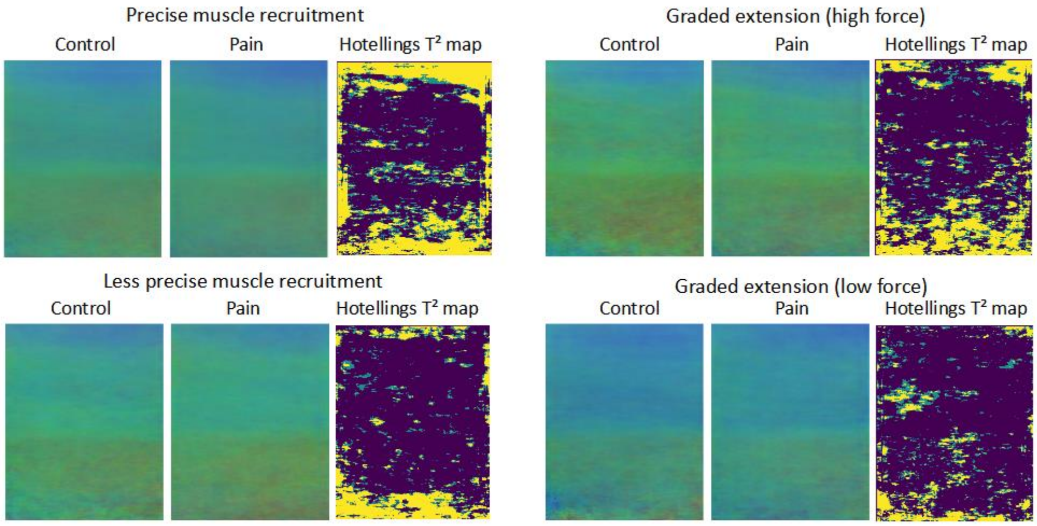

Figure S2. Summary mean images of the elastograms' RGB values; a) Comparison between activities that require precise and less precise neck muscle recruitment; b) Comparison between high force and low force graded extension of the neck muscles; Hotelling's T^2 test map – green color p-value < 0.05 – yellow color p-value < 0.01.

Table S1. The overview of the hyper-parameter grid used for the training of the models. The names of the hyper-parameters are same as in the respective scikit-learn model class.

Models	Hyper-parameters	Values
K-Nearest Neighbors	leaf_size	10, 15, 20, 25
	n_neighbors	3, 5, 7, 9, 11
	weights	uniform, distance
	p	1, 2
Logistic Regression	penalty	l1, l2, elasticnet
	C	1, 10, 100
	solver	liblinear, saga
	max_iter	300
Naïve Bayes	var_smoothing	1e-11, 1e-10, 1e-09, 1e-08, 1e-07
Decision Tree	criterion	gini, entropy, log_loss
	splitter	best, random
	max_depth	50, 100, None
Support Vector Machine	kernel	rbf, poly, sigmoid
	C	1, 10, 100
	gamma	auto, scale
	coef0	0.0, 0.2
Random Forest	n_estimators	1000
	criterion	gini, entropy, log_loss
	max_depth	50, 100, None
	max_features	sqrt, log2
